# Palliative Care Symptoms, Outcomes, and Interventions for Chronic Advanced Patients in Spanish Nursing Homes with and without Dementia

**DOI:** 10.3390/ijerph17051465

**Published:** 2020-02-25

**Authors:** Daniel Puente-Fernández, Concepción Petra Campos-Calderón, Ana Alejandra Esteban-Burgos, César Hueso-Montoro, Concepción Beatriz Roldán-López, Rafael Montoya-Juárez

**Affiliations:** 1 Doctoral Programme in Clinical Medicine and Public Health, University of Granada, 18012 Granada, Spain; danielpuentefdz@correo.ugr.es; 2 Alicante Biomedical Research Institute (ISABIAL), 03001 Alicante, Spain; 3 Department of Nursing, Faculty of Health Sciences, University of Granada, 18016 Granada, Spain; anaestebanburgos@ugr.es (A.A.E.-B.); cesarhueso@ugr.es (C.H.-M.); rmontoya@ugr.es (R.M.-J.); 4 Mind, Brain and Behaviour Research Institute, University of Granada, 18071 Granada, Spain; 5 Department of Statistics and Operational Research, Faculty of Medicine, University of Granada, 18016 Granada, Spain; iroldan@ugr.es

**Keywords:** validation studies, nursing, nursing students, palliative care, self-efficacy

## Abstract

The aim of this study was to compare the symptomatology, palliative care outcomes, therapeutic procedures, diagnostic tests, and pharmacological treatments for people with dementia (PWD) and without dementia (PW/OD) admitted to Spanish nursing homes. Design: This was a cross-sectional study which is part of a long-term prospective follow-up of elderly people performed in nursing homes to measure end-of-life care processes. Participants: 107 nursing home patients with advanced or terminal chronic diseases were selected according to the criteria of the Palliative Care Spanish Society. Setting: Two trained nurses from each nursing home were responsible for participant selection and data collection. They must have treated the residents and had a minimum seniority of 6 months in the nursing home. Measurements: Sociodemographic data; Edmonton Symptom Assessment Scale; Palliative Care Outcome Scale; and prevalence of diagnostic tests, pharmacological treatments, and therapeutic procedures were evaluated. Results: Pain, fatigue, and nausea were found to be significantly higher in the nondementia group and insomnia, poor appetite, and drowsiness were significantly higher in the dementia group. Patient anxiety, support, feeling that life was worth living, self-worth, and practical matters management were higher in the nondementia group. Regarding drugs, use of corticoids was higher in the nondementia group, while use of anxiolytics was higher in the dementia group. Diagnostic procedures such as urine analysis and X-ray were higher in the dementia group. Conclusions: Differences in symptom perception, diagnostic tests, and pharmacological procedures were found between patients with and without dementia. Specific diagnostic tools need to be developed for patients with dementia.

## 1. Introduction

Dementia is one of the main causes of disability and death in the elderly, affecting 35.6 million people worldwide and steadily increasing [1]. Dementia currently affects around 10 million people in the European Region, and its prevalence is expected to double by 2030. There is no cure for dementia, and its development eventually leads to a loss of cognitive and physical functions.

The probability of institutionalization increases significantly with occurrence of dementia (odds ratio (OR) = 154.1) [2]. According to a recent review, poorer cognition, lack of community resources, and uncontrolled behavioral and psychological symptoms are factors that have been linked to institutionalization of older adults with dementia [3].

Because of that, there is a high prevalence of dementia in nursing homes among European countries. A German study published in 2014 showed that the prevalence of dementia was 51.8% in nursing home residents versus 2.7% in community-dwelling elderly [4]. Dementia prevalence was even higher (77.0%) in a study conducted in East London [5].

In Spain, the functions attributed to hospices are assumed by other long-stay centers. In the case of elderly patients, these functions are carried out mostly by nursing homes. Of all Spanish nursing homes, 71.53% were private in 2019, for a total of 3844 private nursing homes. Private centers generally offer a number of places arranged with the Public System [6]. Depending on the number of beds, case complexity, and the legislation of each State, services are offered. In Andalusia (southern Spain), for example, only nursing homes with over 60 beds are required to offer 24 h nursing services and their own medical care [7].

In a three-year prospective study in northern Spain on a cohort of patients with palliative needs, it was observed that almost a quarter of people with palliative needs live in nursing homes [8]. This population is mainly female and elderly, and the prevalent disease is dementia.

Nevertheless, research has demonstrated that in nursing homes, there is a high prevalence of poorly controlled symptoms, such as urinary and fecal incontinence, psychological and behavioral symptoms, pain, nausea, infections, restlessness, dyspnea, edema, and delirium [9,10,11,12].

A palliative care approach is needed for older people with dementia (PWD) institutionalized in nursing homes. High-quality palliative care in nursing homes requires structured evaluation and treatment of physical, psychosocial, emotional, and spiritual symptoms, as well as recognition of and satisfaction with the information needs of patients with dementia and their relatives.

One of the pillars of the palliative approach is to reduce burdensome interventions that might not have value at end of life and to focus on optimizing quality of life. Despite this, aggressive therapies, prescription of futile drugs, and burdensome interventions are still commonplace in older nursing home residents with advanced dementia [13,14]. Health professionals often face dilemmas regarding whether the time has come to not start or to withdraw treatments and interventions because they do not add quality of life [15]. A study conducted in the Netherlands [16] recommended that professionals should respond early to palliative care needs and encourage discussions with patients with dementia and their families about how to deal with therapeutic procedures and drug therapy at the end of life.

Nevertheless, differences in palliative care outcomes, symptoms, and interventions between residents with or without dementia who potentially require palliative care have not yet been examined. Furthermore, no studies have been found that quantitatively evaluate these outcomes in residents with or without dementia in the Spanish context.

We hypothesized that patients without dementia (PW/OD) would have different symptoms and palliative outcomes, consume different drugs, and undergo different diagnostic and therapeutic procedures than patients with dementia.

Related to symptoms, our hypothesis was that patients without dementia would experience more frequently and more intensely symptoms requiring self-identification as pain, fatigue, anorexia, nausea, depression, or anxiety than patients with dementia, while other symptoms would be experienced equally by both groups of patients.

Concerning palliative outcomes, the starting hypothesis was that patients with dementia would present worse outcomes in those aspects related to communication and self-esteem, as well as family anxiety, than those patients without dementia. Patients with dementia would consume more psychopharmaceuticals (anxiolytics, hypnotics, and barbiturates) and fewer analgesics, antiemetics, and antidepressants than patients without dementia due to the limited assessment of pain and other patient-referred symptoms in patients with cognitive impairment. On the other hand, we hypothesized that the use of antibiotics would be higher in patients with dementia. The use of diagnostic procedures would be higher in patients with dementia due to the difficulty in diagnosing concurrent pathologies. On the contrary, the use of therapeutic procedures would be lower in patients with dementia than in the rest of the patients.

The aims of this study were (i) to describe symptoms perceived by professionals, palliative care outcomes, pharmacological treatments, diagnostic tests, and therapeutic procedure interventions and of older adults identified as palliative care patients in Spanish nursing homes and (ii) to compare existing differences between palliative care patients with dementia and those without dementia.

## 2. Materials and Methods

### 2.1. Design

A cross-sectional study was carried out in 6 nursing homes in the Metropolitan Health District of Granada.

### 2.2. Setting and Participants and Data Collection Procedure

Nursing homes were selected based on their institutional characteristics. All had more than 60 beds and were privately funded. The selection of the nursing homes was performed as a convenience selection based on the presence of a multidisciplinary team, professionals interested in the possible involvement in the study, and the offer of both public and private beds. The research team explained the aims of the study to nursing homes managers. In each center, one or two nurses with close knowledge of the residents and who had been working at the nursing home for at least 6 months were responsible for data collection. All the nurses who participated gave their informed consent. In order to control bias and to produce reliable data for the research, these professionals received a training course designed to explain the study in order to ensure uniformity of data collection. The research team was in contact with them by phone if new questions or scenarios were presented and they visited the centers regularly, at least once a month.

Each nursing home professional selected patients with chronic diseases that met the following criteria according to the Spanish Society of Palliative Care (“Sociedad Española de Cuidados Paliativos”—SECPAL):

- Advanced, progressive, and incurable disease
- Lack of reasonable possibilities of response to a specific treatment
- Presence of numerous problems or intense, multiple, multifactorial, and changing symptoms
- Great emotional impact on patient, family, and staff
- Life expectancy limited to 6 months.

Twenty patients meeting the above inclusion criteria were selected from each center for randomized follow-up. They were observed and the data of interest were recorded without interfering with the natural course of events. Data were collected between June 2016 and January 2017. All participants, patients, and caregivers were fully informed and signed the informed consent agreement. Informed consent was given by the patient if he or she did not have dementia and by the patient’s representative in the case of patients with dementia. Thirteen patients dropped out of the study: 3 of them moved on to another nursing home and 10 did not provide informed consent by either the patient or the representative. Among these 10, 6 had dementia and 4 did not.

### 2.3. Instruments

A structured questionnaire was used to collect sociodemographic (age, gender, years in the center) and clinical (medical diagnosis, Charlson Comorbidity Index) data from patient records.

For the systematic symptom assessment, we used the Edmonton Symptom Assessment System (ESAS) [17], the Spanish version of which has been validated in palliative care settings and is easy to complete and to interpret. The ESAS has been validated for both patient and caregiver reports in different settings, including those with older people with multiple morbidities [18]. ESAS was used regularly in all the nursing homes that participated in the study for symptom assessment.

Palliative care outcomes were measured with the Palliative Outcome Scale (POS). The POS is a 10-item multidimensional scale that covers physical, psychological, emotional, and spiritual domains of life. It is a suitable instrument for evaluating palliative care needs coverage and symptoms of people with or without dementia [19]. The Spanish version of the POS [20] was found to be internally consistent (patient version α = 0.64, staff version α = 0.62). The Spanish POS correlated with emotional function and quality of life scales. For dementia patients, ESAS and POS were assessed by trained professionals.

An ad hoc questionnaire was used to identify whether certain interventions had been performed (at least once) in the past month. We evaluated interventions such as common drug prescriptions at end of life (nonopioid analgesics, opioid analgesics, antibiotics, bronchodilators, corticoids, antiemetics, antihistamines, antidepressants, anxiolytics, and hypnotics/barbiturates), diagnostic tests (urine analysis, spirometry, electrocardiograms, X-rays, endoscopy, blood cultures, ultrasound, gammagraphs, electrocardiograms, blood analysis, and gasometry), and therapeutic procedures (urinary catheter, nasogastric catheter, peripheral catheter, enteral nutrition, aerosol sprays, and oxygen therapy) which were used in the past month.

### 2.4. Data Analysis

A descriptive analysis was carried out to describe the main characteristics of the study population. Numerical variables were described by medians and interquartile ranges (P25–P75) and categorical variables by absolute frequencies and percentages.

Quantitative variables were assessed for normality using the Kolmogorov–Smirnov test and all were found to deviate significantly from the normal distribution (*p* < 0.01). Because of this, nonparametric inferential tests were used.

A chi-squared test was used to assess independence between categories and the Mann–Whitney *U* test was used to assess differences between two independent samples. Statistical analysis was performed using IBM SPSS v.25© (IBM Corporation, Armonk, New York, United States); *p*-values of less than 0.05 were considered significant. OR and 95% confidence interval were calculated for each variable in which statistically significant differences were found between the two groups.

### 2.5. Ethical Considerations

All participants (PW/OD) or representatives (PWD) gave their informed consent. The study received the approval of the Granada Research Ethics Committee (PI-0619-2011). In compliance with Spanish law (Article 16, Law 41/2002), patients’ data were anonymized.

## 3. Results

### 3.1. Participants

Overall, 120 patients were selected, but 13 of them were lost for various reasons, such as transfers to other centers. A total 107 nursing home residents were assessed. Residents’ mean age was 84 (81–89) years, and 63.6% of the residents were women. The median length of the nursing home stay was 2.0 years. Chronic Obstructive Pulmonary Disease (COPD) had a significantly higher prevalence among PW/OD than in the PWD group (*p* = 0.05). Other sociodemographic and clinical data of the sample are shown in Table 1.

### 3.2. Perception of Symptom Intensity

In terms of end-of-life-related symptoms evaluated through the ESAS (Table 2), the most intense symptom for the group with dementia was dyspnea ($\tilde{x}$ = 7; range = [4,5,6,7,8,9]), followed by drowsiness ($\tilde{x}$ = 6; range = [4,5,6,7,8]), while for the group without dementia, the most intense symptoms were fatigue ($\tilde{x}$ = 7; range = [5,6,7,8]) and depression ($\tilde{x}$ = 7; range = [3,4,5,6,7,8]). In both groups, the most prevalent symptom was pain. Statistically significant differences were observed in pain (*p* = 0.002), fatigue (*p* = 0.025), nausea (*p* = 0.035), poor appetite (*p* = 0.004), and insomnia (*p* = 0.042), with higher scores in the group without dementia. Drowsiness (*p* = 0.002) was rated higher in the dementia group. No differences in total scale score were observed when comparing groups (Table 2).

### 3.3. Palliative Care Outcomes

Regarding palliative care outcomes, the mean score of the global POS of the group with dementia ranked between one and two, while those of the group without dementia ranked between two and three. More intense anxiety (*p* = 0.05) and depression (*p* = 0.05) were observed in the nondementia group compared with the dementia group. As shown in Table 3, the group with dementia spent more time waiting or repeating tests than the group without dementia (*p* = 0.49). However, the group without dementia showed higher scores for sharing their feelings with their family (*p* = 0.001) and more practical matters (*p* = 0.002) than patients with dementia. As for the Eastern Cooperative Oncology Group (ECOG), patients in the dementia group were more dependent than those in the nondementia group (*p* = 0.001). The total score was lower in the group of patients with dementia.

### 3.4. Pharmacological Treatments, Diagnostic Tests, and Therapeutic Procedures

The most commonly used drugs for patients with dementia were hypnotics and barbiturates (62.7%), followed by analgesics (60%) and antidepressants (33%). For the group without dementia, 60.7% received nonopioid analgesics, followed by anxiolytics and bronchodilators (32.1%). Statistically significant differences were observed for corticoids (*p* = 0.035) and anxiolytics (*p* = 0.005). The percentage of patients with corticosteroids was higher in the nondementia group, while the percentage of patients with anxiolytics was higher in the dementia group.

Patients with dementia showed a higher probability of taking anxiolytics than those without dementia (OR = 3.521). Patients with dementia showed a lower probability of taking corticoids than those without dementia (OR = 0.306), as shown in Table 4.

Regarding the diagnostic tests, almost half of the patients in the dementia group (45.1%) had a urine test and 38.3% had a blood test. On the other hand, in the group without dementia, 32.7% had a blood analysis and 19.6% had a urine analysis. It is notable that in the case of X-rays, in the group with dementia, 17.5% were performed, while in the group without dementia, no case was reported. Greater use of urine analysis (*p* = 0.009) and X-rays (*p* = 0.009) was observed in patients with dementia compared with patients without dementia (Table 5).

Patients with dementia showed 3.356 (1.422; 7.937) and 25.393 (1.430; 446.200) times the probability of having a urinalysis and X-ray performed than those without dementia, respectively.

For the group with dementia, 25.5% had an IV peripheral catheter and 23.7% carried a urinary catheter. Only 19.6% and 15.7% had oxygen therapy and aerosol sprays, respectively. For the group without dementia, the most frequent interventions were oxygen therapy (39.3%) and aerosol sprays (32.1%). The use of oxygen therapy was significantly less frequent among patients with dementia (OR = 0.378) (Table 6).

## 4. Discussion

Intensity of perceived symptoms, diagnostic and therapeutic procedures, and quality of palliative care were found to differ between residents with or without dementia in Spanish nursing homes. Our results showed that pain, fatigue, and nausea were significantly higher in the nondementia group. On the other hand, insomnia, poor appetite, and drowsiness were significantly higher in the dementia group. Concerning drugs, use of corticoids was higher in the nondementia group, while use of anxiolytics was higher in the dementia group. Diagnostic procedures such as urine analysis and X-ray were higher in the dementia group. Patient anxiety, lack of perceived support, lack of self-worth, and lack of practical matters management were higher in the nondementia group. Below, the implications for practice and research are discussed.

Although no differences were observed between groups in terms of total ESAS scale score, significant differences were observed between different symptoms. Pain is one of the most studied symptoms in residents with dementia institutionalized in nursing homes [11,12,21,22,23]. As our results highlighted, this symptom is usually one of the most prevalent in advanced stages and at the end of life [11]. Despite this, there is great difficulty in assessing pain in dementia patients because nonverbal signs of pain can be misinterpreted as neuropsychiatric or behavioral symptoms [11,21,23]. Smedbäck et al. [11] pointed out the need to develop specific scales for patients with dementia to help assess this and other symptoms.

According to our hypothesis and previous studies [12,24,25], other self-reported symptoms such fatigue, nausea, poor appetite, and insomnia were rated higher in nondementia patients. As it happens with pain, this may be linked to a lack of assessment tools [11,26] rather than a real low prevalence/intensity of these symptoms in dementia patients [24].

Regarding palliative care outcomes, contrary to our initial hypothesis, the mean score of the POS was higher in nondementia patients, who also showed more anxiety, a lack of family support, and felt worst about themselves than dementia patients. Again, this could be linked to the absence of specific validated tools in the Spanish context to report anxiety and depression in dementia patients. As with the ESAS, POS data of dementia patients were reported by professionals. Nevertheless, professionals of nursing homes are not well trained in recognizing and treating emotional problems in dementia patients [12,27].

Although family anxiety was found to be higher in dementia patients, this difference was not statistically significant. It could be expected that families of dementia patients in nursing homes report higher levels of anxiety [24,28]. However, the anxiety of family members is high in dementia and nondementia patients, especially when faced with situations related to end-of-life decision-making [29,30].

According to our results, having dementia does not seem to be an impediment to receiving information, compared to those without dementia. It is necessary to highlight that the POS includes all the information that has been given both to the patient or the family. Communication is a challenge for dementia patients’ relatives, but it is not only for them. In fact, Hermans et al. [31] showed in their results that patients with dementia received less information and received less support from their families than the patients in the group without dementia. In our study, there were no statistically significant differences for given information between groups. Differences found between Hermans et al. [31] and our results may be due to different cultural patterns in dealing with institutionalized patients [32].

On the other hand, professionals and families spend more time on appointments related to healthcare. This may be due to greater difficulty in diagnosing pain or other symptoms and their possible causes [21].

In terms of the drugs used, contrary to the initial hypothesis, although more pain was reported in patients without dementia than in patients with dementia, no differences were observed in analgesic consumption. This absence of differences may indicate that pain is underevaluated and undertreated in nursing homes [23,33,34]. However, it is difficult to know if patients are receiving enough analgesia, even in nondementia patients [23]. In a study carried out in Polish nursing homes, only 30% of residents with moderate or severe cognitive impairment were treated with analgesics. Most of them received medication from the first step of the analgesic scale, and only a few received opioids even if they experienced moderate pain [23].

Physicians have shown a preference to start with a weak analgesic, such as a Nonsteroidal Anti-Inflammatory Drug (NSAID), and gradually move upwards, rather than begin with powerful analgesics such as opioids [21]. In our study, although no differences were observed between groups, the percentage of opioid intake was lower than in other studies conducted in nursing homes with similar samples. Hendriks et al. [9] found that pain was recognized in 73% of patients, but in this study, patients were evaluated only in the last week of life, where pain is more prevalent and intense.

Regarding antibiotic consumption, a study carried out by van den Noortgate et al. [33] in Belgian nursing homes observed the last 48 h of life in these institutions. In this study, 63.6% of the patients consumed antibiotics.

In our results, contrary to the initial hypothesis, no statistically significant difference was found in antibiotic consumption between groups, as opposed to what was observed by Hendriks et al. [16] in their study, which showed greater antibiotic intake in patients with dementia.

According to our hypothesis, no differences were found in reported anxiety between dementia and nondementia patients, and a greater administration of anxiolytics and hypnotics/barbiturates was observed in the group with dementia. Agitation and restlessness can have several causes, such as pain or emotional distress [21]. Diagnosing the cause in dementia patients is very complicated because most of the time, these patients cannot verbally report these symptoms and this may lead to undertreatment or mistreatment. Other psychotropic drugs such as antipsychotics or barbiturates should be analyzed in further studies.

Due to the difficulties in diagnosing concurrent diseases or complications in dementia patients, according to our hypothesis, higher percentages of diagnostic procedures were observed in these patients for many of the evaluated tests, although statistically significant differences were only found in urine analysis and X-ray tests. The difficulty of diagnosing urinary tract infections has been previously reported in patients with advanced dementia in nursing homes [35]. Similarly, the high prevalence of microfractures in older adults with dementia (men and women, 80 years or more) has been reported in other study [36]. Hommel et al. [36] additionally remarked that these fractures are often underdiagnosed and may be considered as a cause of pain-related signs in dementia patients.

Regarding therapeutic procedures, contrary to the initial hypothesis, there were no differences between groups. This is consistent with the literature, where patients with dementia are not usually subjected to onerous interventions [16]. Nevertheless, we found statistically significant differences between groups for aerosol sprays and oxygen therapy. This may be due to difficulty in evaluation as well as other symptoms such as pain, which is reflected in undertreatment [33,34].

Some limitation should be pointed out. For this study, palliative patients were identified according to current Spanish Society of Palliative Care (“Sociedad Española de Cuidados Paliativos”—SECPAL) criteria, but since the data collection procedure finished, specific tools such as the NECPAL-CCOMS-ICO © tool [37] have been developed and improved to assess palliative needs of advanced chronic patients, like those selected for this study, in the Spanish context. Nevertheless, the SECPAL criteria were the most widely used criteria for the Spanish population at the beginning of data collection. On the other hand, as it has been pointed out in the discussion section, although the ESAS and POS have been used for dementia patients before [19], it is necessary to develop and adapt to the Spanish context specific tools to evaluate symptoms and palliative care outcomes in these patients. It should be remembered that data on symptom intensity and prevalence as well as POS items have been reported by professionals. This carries a potential bias, as professionals may over- or underestimate certain aspects.

Finally, sample size may be a limitation for analysis in order to find other statistically significant differences between the two populations. Regardless, our study showed numerous differences with a high level of significance in spite of the sample size. However, the results of this study should be interpreted with caution, as they refer to a specific geographical context. Comparisons between different countries should be made in the future so that the differences found can be generalized.

## 5. Conclusions

Pain, fatigue, and nausea were found to be significantly higher in patients without dementia. In contrast, insomnia, poor appetite, and drowsiness were significantly higher in the dementia patients. Regarding drugs, use of corticoids was higher in the nondementia group, while use of anxiolytics was higher in the dementia group. Diagnostic procedures such as urine analysis and X-ray were higher in the dementia group. Patient anxiety, lack of perceived support, and lack of practical matters management were higher in nondementia patients, while more time was wasted on healthcare appointments in the dementia group. Specific tools are needed in order to evaluate symptoms and palliative care outcomes in dementia patients.

This is one of the first studies to evaluate and compare symptoms, pharmacological treatments, diagnostic and treatment procedures, and palliative outcomes between dementia and nondementia patients in nursing homes. This study provides valuable data about which symptoms are more prevalent and intense in dementia and nondementia patients, which are the most performed procedures, and which are the unmet palliative care needs.

## Figures and Tables

**Table 1:** Sociodemographic and clinical characteristics of the patients.

Variables	Total Sample (*n* = 107)		PWD (*n* = 51)		PW/OD (*n* = 56)		*p*
**Age, md (P25–P75)**	84	(81–89)	85	(82–92)	83.5	(77–89)	0.142a
**Female, *n***(%)	68	(63.6)	33	(64.7)	35	(62.5)	0.972a
**Years in the center, md (P25–P75)**	2	(1–4)	2	(1.7–5)	2	(1–4)	0.500b
**CCI, md (P25–P75)**	3.5	(2–6)	4	(3–6)	3	(2–6)	0.228a
**Coexisting conditions**							
**Myocardial infarction, *n***(%)	6	(5.6)	3	(6.1)	3	(5.4)	0.866b
**Heart failure, *n***(%)	28	(26.2)	11	(22.4)	17	(30.4)	0.488b
**Peripheral vascular disease, *n***(%)	10	(9.3)	6	(12.2)	4	(7.1)	0.579b
**Thromboembolic disease, *n***(%)	7	(6.5)	3	(6.1)	4	(7.1)	1.000b
**Vascular brain ictus/damage, *n***(%)	45	(42.1)	25	(51.0)	20	(35.7)	0.167b
**Hemiplegia, *n***(%)	14	(13.1)	4	(8.2)	10	(17.9)	0.242b
**Arterial hypertension, *n***(%)	63	(58.9)	28	(57.1)	35	(62.5)	0.719b
**COPD, *n***(%)	25	(23.4)	5	(10.2)	20	(35.7)	0.005b
**Arrhythmia, *n***(%)	21	(19.6)	12	(24.5)	9	(16.1)	0.406b
**Renal disease, *n***(%)	19	(17.8)	8	(16.3)	11	(19.6)	0.852b
**Diabetes, *n***(%)	34	(31.8)	17	(36.2)	18	(32.7)	0.879b
**Malignant tumor, *n***(%)	17	(15.9)	6	(12.2)	11	(19.6)	0.447b
**Solid tumor with metastasis, *n***(%)	10	(9.3)	2	(4.1)	8	(14.3)	0.149b

PWD, patients with dementia; PW/OD, patients without dementia; CCI, Charlson Comorbidity Index; COPD, Chronic Obstructive Pulmonary Disease; a: Mann–Whitney; b: chi-squared; md, median; (P25–P75) interquartile range.

**Table 2:** Comparison of intensity of perceived symptoms using the Edmonton Symptom Assessment System (ESAS) in patients of Nursing Homes with or without dementia.

Symptoms and Concerns	% (Total)	PWD ^a^	PW/OD ^a^	*p* ^b^
**1. Pain**	87.9 (94)	3.5 (2–6)	6 (4–7)	0.002
**2. Fatigue**	82.2 (88)	5 (4–7)	7 (5–8)	0.025
**3. Nausea**	33.6 (36)	3 (2–4)	4.5 (3–6)	0.035
**4. Depression**	61.7 (66)	4 (3–7)	7 (3–8)	0.115
**5. Anxiety**	62.6 (67)	5 (4–6)	5.5 (4–8)	0.184
**6. Drowsiness**	86.0 (92)	6 (4–8)	4 (2–6)	0.002
**7. Poor appetite**	68.2 (73)	4 (2–6)	6 (5–8)	0.004
**8. Not feeling well**	74.8 (80)	6 (4–7)	6 (5–7)	0.197
**9. Dyspnea**	54.2 (58)	7 (4–9)	6 (2–8)	0.194
**10. Insomnia**	64.5 (69)	3 (2–7)	6 (4–8)	0.042
**TOTAL**	92.5 (99)	3 (2–5)	4 (2–6)	0.613

^a^ median score and interquartile range; ^b^ Mann–Whitney.

**Table 3:** Perception of palliative care outcomes reported by the Palliative Outcome Scale (POS) in patients of NH with dementia (PWD) and without dementia (PW/OD).

Palliative Care Outcomes	% (Total)	PWD	PW/OD	*p* ^a^
1. Has the patient been affected by pain? (from 0 = “Not at all” to 4 = “Overwhelmingly”)	87.9 (94)	1 (1–2)	1 (1–2)	0.069
2. Does the patient’s other symptoms (e.g., nausea, coughing, or constipation) seem to be affecting how well s/he feels? (from 0 = “Not at all” to 4 = “Overwhelmingly”)	74.8 (80)	2 (1–2)	2 (1–2)	0.775
3. Has s/he been feeling anxious or worried about her/his illness or treatment? (from 0 = “Not at all” to 4 = “The patient does not seem to think of anything else”)	59.8 (64)	2 (1–2)	2 (2–3)	0.050
4. Have any of their family or friends been anxious or worried about the patient? (from 0 = “Not at all” to 4 = “Yes, they always seem preoccupied with worry”)	89.7 (96)	3 (2–3)	2 (2–3)	0.111
5. How much information has been given to the patient and his/her family or friends? (from 0 = “Full information or as much as wanted” to 4 = “None at all”)	83.2 (89)	3 (3–4)	4 (3–4)	0.232
6. Has the patient been able to share how s/he is feeling with family or friends? (0 = “Yes, as much as s/he wanted to” to 4 = “No, not at all with anyone”)	60.7 (65)	1.5 (1–3)	3 (2–4)	0.001
7. Do you think the patient has felt good about his/herself? (from 0 = “Yes, all the time” to 4 = “No, not at all”)	52.3 (56)	2 (1–2)	2 (1–4)	0.050
8. Do you think s/he felt life was worth living? (from 0 = “Yes, all the time” to 4 = “No, not at all”)	60.7 (65)	2 (1–2)	2 (1–3)	0.065
9. How much time do you feel has been wasted on appointments relating to the healthcare of this patient (e.g., waiting around for transport or repeating tests)? (from 0 = “None at all” to 4 = “More than half a day wasted”)	85.0 (91)	2 (2–2)	2 (1–2)	0.049
10. Have any practical matters resulting from his/her illness, either financial or personal, been addressed? (from 0 = “Practical problems have been addressed and his/her affairs are as up to date as s/he would wish” to 4 = “Practical problems exist which were not addressed”)	45.8 (49)	2 (2–3)	3 (2–3)	0.002
11. Eastern Cooperative Oncology Group (ECOG)	100.0 (107)	3 (3–4)	2 (1–3)	0.001
Total	96.3 (103)	18 (15–21)	20 (18–24)	0.022

^a^ Mann–Whitney.

**Table 4:** Comparison of pharmacological treatments in patients of NH with dementia (PWD) and without dementia (PW/OD).

Pharmacological Treatments	PWD (*n* = 51)	PW/OD (*n* = 56)	*p* ^b^	OR (95% CI)
Nonopioid analgesics, (%)	60.0	60.7	1.000	
Opioid analgesics, (%)	14.9	16.4	1.000	
Antibiotics, (%)	23.5	21.4	0.978	
Bronchodilators, (%)	23.5	32.1	0.438	
Corticoids, (%)	11.8	30.4	0.035	0.306 (0.109; 0.853)
Antiemetics, (%)	9.8	8.9	1.000	
Antihistamines, (%)	9.8	8.9	1.000	
Antidepressants, (%)	33.3	32.1	1.000	
Anxiolytics, (%)	49.0	21.4	.005	3.521 (1.520; 8.198)
Hypnotics/barbiturates, (%)	62.7	32.1	.839	

^b^ chi-squared; OR (95% CI), odds ratio (95% confidence interval of the odds ratio).

**Table 5:** Comparison of diagnostic tests in patients of NH with dementia (PWD) and without dementia (PW/OD).

Diagnostic Tests	PWD (*n* = 51)	PW/OD (*n* = 56)	*p* ^b^	OR (95% CI)
**Urine analysis, (%)**	45.1	19.6	0.009	3.356 (1.422; 7.937)
**Spirometry,** (%)	2.0	3.6	1.000	
**Electrocardiograms,** (%)	3.0	3.6	1.000	
**X-rays,** (%)	17.6	0.0	0.003	25.393 (1.430; 446.200)
**Endoscopy,** (%)	3.9	0.0	0.225	
**Blood cultures,** (%)	5.9	0.0	0.105	
**Ultrasound,** (%)	5.9	3.6	0.668	
**Gammagraphs,** (%)	2.0	0.0	0.477	
**Electrocardiograms,** (%)	19.1	14.5	0.722	
**Blood analysis,** (%)	38.3	32.7	0.705	
**Gasometry,** (%)	9.8	3.6	0.254	

^b^ chi-squared (Fisher’s exact test); OR (95% CI), odds ratio (95% confidence interval of the odds ratio).

**Table 6:** Comparison of therapeutic procedures in patients of NH with dementia (PWD) and without dementia (PW/OD).

Therapeutic Procedures	PWD (*n* = 51)	PW/OD (*n* = 56)	*p* ^b^	OR (95% CI)
**Urinary catheter,** (%)	23.7	13.2	0.176	
**Nasogastric catheter,** (%)	19.6	10.7	0.309	
**IV peripheral catheter,** (%)	25.5	26.8	1.000	
**Enteral nutrition,** (%)	15.7	7.1	0.275	
**Aerosol sprays,** (%)	15.7	32.1	0.079	
**Oxygen therapy,** (%)	19.6	39.3	0.045	0.378 (0.329; 0.994)

^b^ chi-squared; OR (95% CI), odds ratio (95% confidence interval of the odds ratio).
